# 

**Published:** 1875-03-15

**Authors:** 


*ORIGINAL COMMUNICATIONS:*

The Difference between Carcinoma and Sarcoma. 147

Cerebral Abscess.—History—Death—Autopsy. By D. A. K. Steele, M.D. ...... 150

*EDITORIAL DEPARTMENT:*

Woman's Hospital Medical College; American Medical Association. ...... 152

*CORRESPONDENCE:*

A Morning in the Charite Hospital. ...... 153

*GLEANINGS FROM OUR EXCHANGES:*

Ætiology and Treatment of Pneumonia. ...... 154

The Contagion of Puerperal Fever. ...... 156

Clinical Lecture on the Use of Digitalis in Diseases of the Heart. By H. C. Wood, Jr., M.D. 158

Interesting Experiments upon the Body of Heidenblut the Murderer. ...... 159

Raw Cotton Dressing for Wounds. By Prof. Wm. H. Van Buren, M.D. ...... 161

*BOOK REVIEWS:* ...... 174

